# Propionate supplementation promotes the expansion of peripheral regulatory T-Cells in patients with end-stage renal disease

**DOI:** 10.1007/s40620-019-00694-z

**Published:** 2020-03-06

**Authors:** Fabian Meyer, Felix S. Seibert, Mikalai Nienen, Marius Welzel, Daniela Beisser, Frederic Bauer, Benjamin Rohn, Timm H. Westhoff, Ulrik Stervbo, Nina Babel

**Affiliations:** 1 Medical Department I , Centre for Translational Medicine, Marienhospital Herne, Universitätsklinikum Der Ruhr-Universität Bochum, Ruhr-Universität Bochum, Hölkeskampring 40, 44623 Herne, Germany; 2 Biodiversity, University of Duisburg-Essen, Essen, Germany; 3 Charité, Universitätsmedizin Berlin, Corporate Member of Freie Universität Berlin, Humboldt-Universität Zu Berlin, and Berlin Institute of Health, Berlin-Brandenburg Center for Regenerative Therapies, Berlin, Germany

**Keywords:** Short-chain fatty acids, Regulatory T-cells, End-stage renal disease, Propionate

## Abstract

Patients with end-stage renal disease (ESRD) suffer from a progressively increasing low-grade systemic inflammation, which is associated with higher morbidity and mortality. Regulatory T cells (Tregs) play an important role in regulation of the inflammatory process. Previously, it has been demonstrated that short-chain fatty acids reduce inflammation in the central nervous system in a murine model of multiple sclerosis through an increase in tissue infiltrating Tregs. Here, we evaluated the effect of the short-chain fatty acid propionate on the chronic inflammatory state and T-cell composition in ESRD patients. Analyzing ESRD patients and healthy blood donors before, during, and 60 days after the propionate supplementation by multiparametric flow cytometry we observed a gradual and significant expansion in the frequencies of CD25^high^CD127^−^ Tregs in both groups. Phenotypic characterization suggests that polarization of naïve T cells towards Tregs is responsible for the observed expansion. In line with this, we observed a significant reduction of inflammatory marker CRP under propionate supplementation. Of interest, the observed anti-inflammatory surroundings did not affect the protective pathogen-specific immunity as demonstrated by the stable frequencies of effector/memory T cells specific for tetanus/diphtheria recall antigens. Collectively, our data suggest that dietary supplements with propionate have a beneficial effect on the elevated systemic inflammation of ESRD patients. The effect can be achieved through an expansion of circulating Tregs without affecting the protective pathogen-reactive immunity.

**Electronic supplementary material:**

The online version of this article (10.1007/s40620-019-00694-z) contains supplementary material, which is available to authorized users.

## Introduction

Patients with end-stage renal disease (ESRD) frequently suffer from chronic systemic inflammation. This is associated with reoccurring infections, protein-energy-wasting, and cardiovascular events resulting in increased mortality and morbidity [1]. In fact, cardiovascular events are the leading causes of death in ESRD [2]. The reasons for the chronic inflammation are multifactorial and encompass oxidative stress, hyperazotemia, intestinal dysbiosis, acidosis, decreased cytokine elimination, and frequent infections [3, 4].

Recognizing the crucial role of dysregulated inflammation in the pathogenesis of numerous diseases, different approaches have been applied to revert to the imbalance between pro- and anti-inflammatory mechanisms, which underlie the process of chronic inflammation [5]. Thus, a very recent study reported on a reduction of pro-inflammatory parameters under the alimentary supplementation with short-chained fatty acids (SCFA) in hemodialysis patients [6]. The major compound of SCFA, sodium propionate, led to a significant decrease of pro-inflammatory parameters CRP, IL-2, and IL-17 with an increase of the anti-inflammatory factors TGF-β and IL-10 and general improvement of quality of life [6].

SCFA are produced in the small intestine and colon by anerobic fermentation of water-soluble dietary fibers. SCFA influence the microbiome and mucosa metabolism and have, in addition to the anti-inflammatory effects, been associated with a positive effect on oxidative stress, insulin sensitivity, and a negative association with the formation of intestinal uremic toxins [6–11]. Propionate and other SCFA are actively adsorbed by the gut epithelium and reach circulation through the hepatic portal vein [12]. The concentration of propionate in human plasma is generally reported to be in the range of 3.4–4.9 µM [13–15], but values of 0.3–13.3 µM has also been reported [11, 16]. Recently, a beneficial effect of dietary supplementation with SCFA was shown in a mouse model for inflammation in the central nervous system (CNS) [17]. The authors found that the amelioration of paralysis was associated with an increase in the frequencies of CD4^+^ regulatory T-cells (Tregs). This effect could at least partially be explained by propionate supplementation.

Treg prevent aberrant immune reactions and are associated with the pathogenesis of several autoimmune disorders [18–21]. Importantly, systemic lupus erythematosus (SLE) and systemic sclerosis, which both can cause ESRD, are associated with functional and quantitative altered Tregs [22–24]. Reduced numbers and impaired suppressive function of Tregs has been observed in ESDR patients with different underlying renal diseases [25].

Based on these findings we hypothesized that dietary supplementation with sodium propionate could have a regulatory effect on the chronic inflammatory state of dialysis patients. We assume that this effect is associated with an enhancement of circulating Tregs. We further hypothesized that this Treg increase will not affect protective pathogen-specific T cell immunity.

## Results

### Demographic characteristics and laboratory data

The study participants were followed for a total of 150 days and separated in the three phases: pre-treatment, treatment, and post-treatment phases (Supplementary Fig. 1). While the pre-treatment phase (day − 60 to 0) served as control to assess the baseline levels, the propionate treatment phase was designed to assess the immune changes during 30 days of treatment with 2 × 500 mg sodium propionate per day. The post-treatment phase assessed the immune cell composition 60 days after the discontinuation of the treatment as the follow-up phase.

Demographic characteristics of ESRD patients are presented in Table 1. Briefly, the mean patient age was 73.8 years, female/male ratio was 2/8, and the mean duration of dialysis treatment was 6.7 months. The average age of the healthy control group was 46.4 with a female/male ratio of 4/3.

**Table 1:** Biochemical laboratory analysis

Marker	Baseline	After propionic acid	Follow-up
Hemoglobin (g/dl)	10.7 ± 0.95	11.03 ± 0.78	10.95 ± 1.11
WBC (10^3^/µl)	5.34 ± 1.3	5.73 ± 1.011	5.54 ± 1.77
Thrombocytes (10^3^/µl)	170.04 ± 52.88	189.66 ± 60.88	189.0 ± 73.001
Creatinine (mg/dl)	7.45 ± 2.01	7.52 ± 1.56	8.23 ± 1.46
Urea (mg/dl)	114.08 ± 31.73	115.0 ± 33.81	110.75 ± 29.14
Na (mmol/l)	136.881 ± 2.53	138.16 ± 1.94	137.88 ± 2.69
K (mmol/l)	5.94 ± 0.27	5.36 ± 0.5	5.23 ± 0.4
Ca (mmol/l)	2.09 ± 0.12	2.13 ± 0.16	2.06 ± 0.15
CRP (mg/dl)	1.22 ± 1.03	0.63 ± 0.39*	1.21 ± 1.29

Shown as mean ± SD

^*^p < 0.05

Routine monitoring of electrolytes, blood cell counts and renal parameters demonstrated stable values during the propionate supplementation and two months after its discontinuation. In contrast, the inflammatory marker C-reactive protein demonstrated a significant reduction of CRP level under supplementation. Two months after therapy discontinuation, CRP values rose to the pre-treatment levels (Table 1).

### No differences in the frequencies of Tregs between dialysis patients and healthy blood donors

**Fig. 1:**
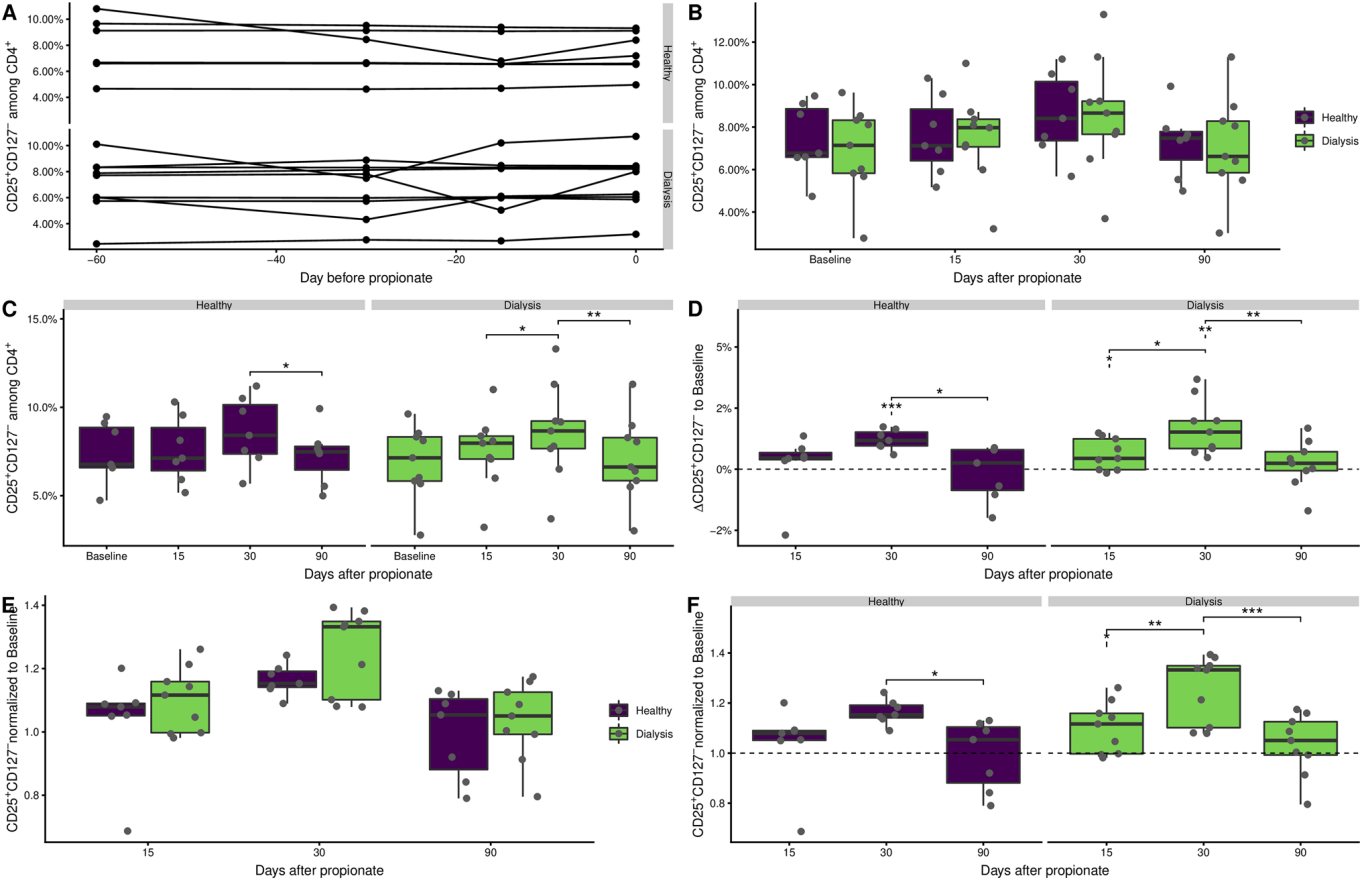
CD25^high^CD127^−^ Tregs expand during propionate supplementation. Cells were analyzed ex vivo by flow cytometry. Tregs were identified as CD25^high^CD127^−^ according to the gating strategy in Supplementary Fig. 2. **a** Frequency of Tregs during the baseline phase of the study. The average over the baseline phase was taken for each study participant and used as a single baseline value. **b** Frequency of Tregs during the propionate supplementation and follow-up phase of the study focusing on differences between study groups. **c** As in **b** but focusing on changes over time. **d** Difference of Treg frequencies over time to the base line. The downward pointing line under asterisks indicates comparison of mean to 0. **e** Ratio of Tregs frequency during the propionate and follow-up phase to the baseline focusing on differences between study groups. **f** As in **e** but with focus on changes over time. The downward pointing line under asterisks indicates comparison of mean to 1. The boxes represent the 25th, 50th, and 75th percentile and the whiskers represent the range of the observations excluding outliers. Each point signifies a single donor. Only significant differences are annotated. Asterisks indicate the p value (*p < 0.05; **p < 0.01; ***p < 0.001)

The four time points of the pre-treatment/baseline phase were used to evaluate the general variation of CD25^high^CD127^−^ Tregs over time in dialysis patients and healthy blood donors (see gating in Supplement 1). No particular fluctuation could be observed for any participant (Fig. 1a). Of interest, no significant differences were found in the frequencies of Tregs between dialysis patients and healthy blood donors. The mean over the entire baseline phase was 7.4% (range 4.7–9.5%) for the healthy donors and 6.9% (range 2.8–9.6%) for the dialysis patients. Similarly, no significant differences between the two groups were found in follow-up. Over all during the three phases of the study, the Treg frequency in the two groups was similar (Fig. 1b).

### Propionate supplementation causes expansion of Tregs in peripheral blood

In both groups, an apparent increase in Tregs during the propionate treatment phase could be observed when compared to the average at the baseline-phase (Fig. 1c). This was particularly pronounced for the dialysis patients, where the frequencies of Tregs significantly increased from day 15 to day 30. The expansion of Tregs during the treatment phase was followed by a significant decrease of Tregs in post-treatment phase where we observed Treg frequencies comparable to those of the baseline phase for both groups. Statistically significant changes before, during, and after the treatment were observed for Treg frequencies (Fig. 1c), as well as for the fold change compared to the baseline frequencies (Fig. 1d). Here, we additionally noted a large ratio at day 30 for the dialysis patients compared to the healthy donors (Fig. 1e, f).

Taken together, the frequencies of Tregs increase during dietary supplementation with propionate. The dynamics of the change appear faster for dialysis patients compared to healthy donors.

### Functionality of conventional pathogen-reactive T-cells remains unaffected

Given the immune regulatory effects of Tregs we were wondering if the observed increase in Tregs could affect the function of non-regulatory T-cells. Therefore, the recall antigen-reactive immunity was evaluated. To this end, blood was stimulated with tetanus/diphtheria vaccine and the specific reactivity to the recall antigen was measured (Supplementary Fig. 3). No clear systematic changes in the frequency of granzyme B (GrzB)-, interferon gamma (IFN-γ)-, interleukin (IL)-2- and IL-17-, and tumor-necrosis-factor alpha (TNF-α)- producing CD4^+^ (Supplementary Fig. 3a) and CD8^+^ (Supplementary Fig. 3b) T-cells between the three study phases were observed. Similarly, to the frequencies of Tregs, no differences in frequencies of antigen-reactive conventional T cells were observed between healthy donors and dialysis patients after propionate treatment.

Taken together, our data demonstrate that propionate supplementation does not affect antigen-specific memory T- cell response.

### Treg subpopulations do not change during propionate supplementation

**Fig. 2:**
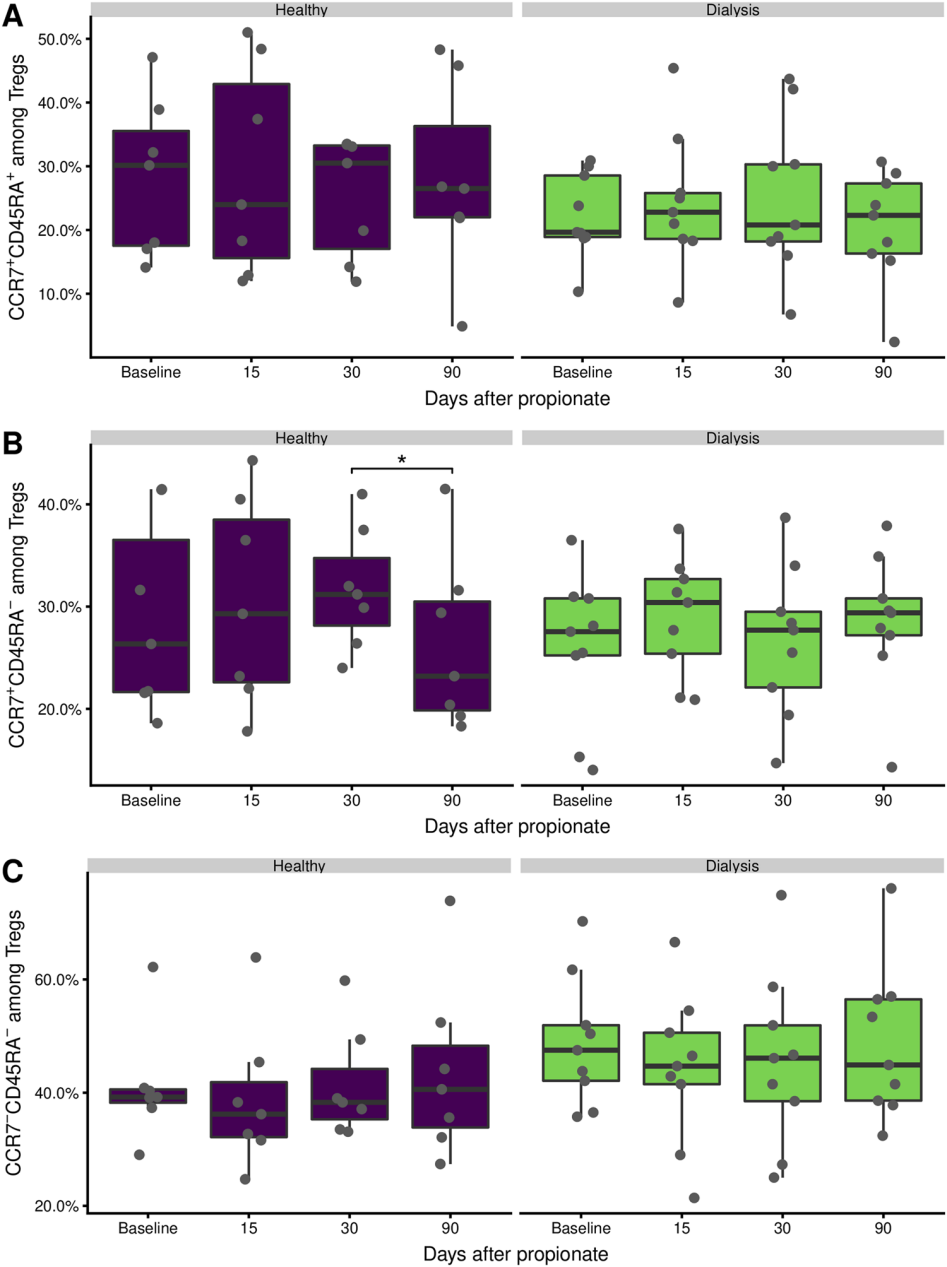
Treg subpopulations are not altered during propionate supplementation. Tregs and their subpopulations were analyzed ex vivo by flow cytometry according to the gating strategy in Supplementary Fig. 2. **a** CCR7^+^CD45RA^+^ naïve like Tregs. **b** CCR7^+^CD45RA^–^ central memory like Tregs. **c** CCR7^−^CD45RA^–^ effector memory like Tregs. The boxes represent the 25th, 50th, and 75th percentile and the whiskers represent the range of the observations excluding outliers. Each point signifies a single donor. Only significant differences are annotated. Asterisks indicate the p value (*p < 0.05; **p < 0.01; ***p < 0.001)

Given the increased number of Tregs under sodium propionate supplementation, we were wondering what mechanism underlies the observed Tregs expansion. T cells can be divided into naïve and memory subpopulations by their expression of the chemokine receptor 7 (CCR7) and CD45RA [26, 27]. As for conventional T-cells, naïve Treg cells are marked by CCR7^+^CD45RA^+^, central memory Treg by CCR7^+^CD45RA^–^, and effector memory Treg by CCR7^−^CD45RA^–^. Following these subpopulations overtime, we observed some minor, but insignificant, fluctuations (Fig. 2). We did observe a significant decline in the CCR7^+^CD45RA^–^ central memory among the healthy donors from the end of the propionate phase at day 30 to the follow-up at day 90 (Fig. 2b). This decline was not accompanied by a corresponding increase in any of the other subpopulations (Fig. 2a–c). It does nonetheless indicate a slight shift to the central memory in the healthy. Taken together, the naïve and memory Treg subpopulations remain unperturbed in peripheral blood under propionate supplementation.

### The proliferation rate of regulatory T-cells is unaffected

**Fig. 3:**
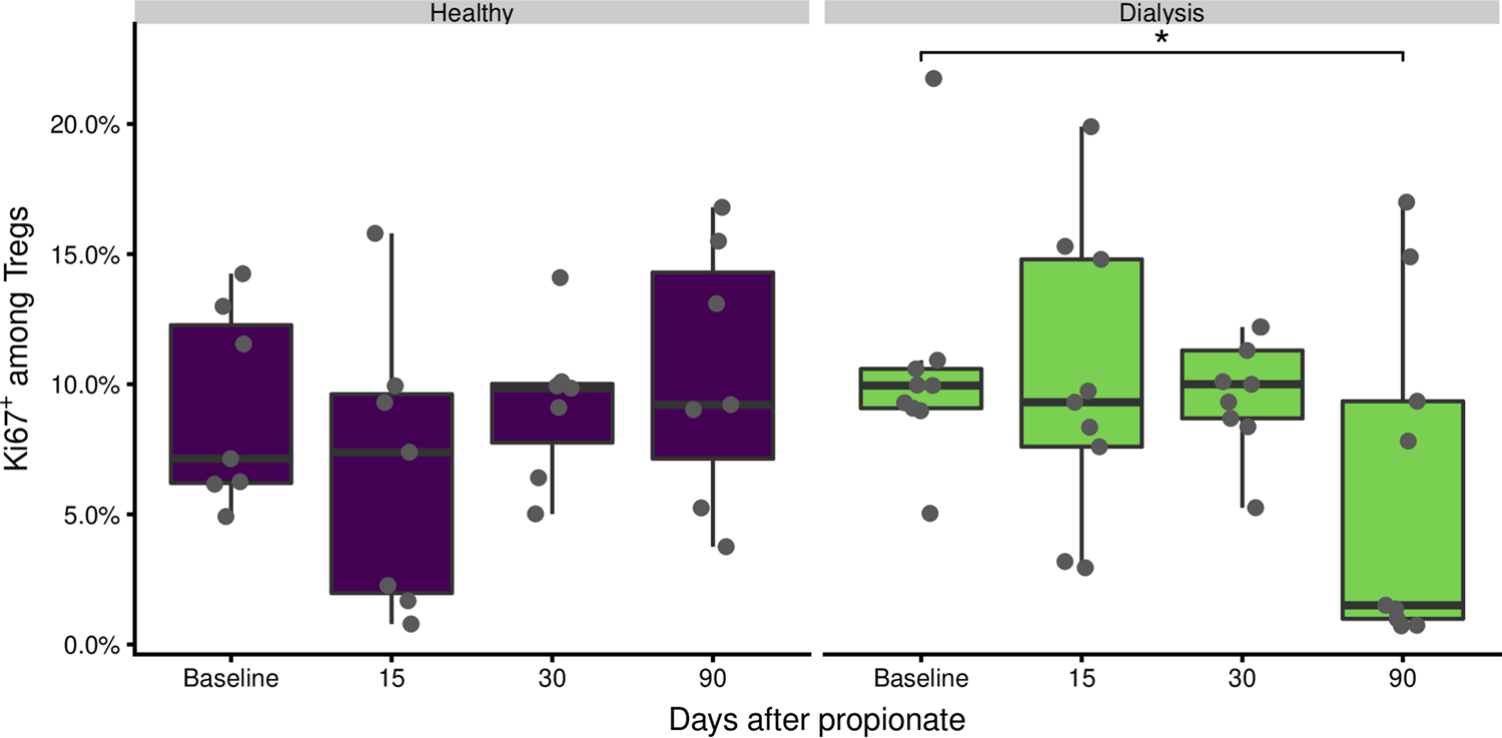
Proliferative capacity of Tregs is not modified by propionate intake. Proliferating CD25^high^CD12^7−^ Tregs were identified by KI76-expression according to the gating strategy in Supplementary Fig. 2. The boxes represent the 25th, 50th, and 75th percentile and the whiskers represent the range of the observations excluding outliers. Each point signifies a single donor. Only significant differences are annotated. Asterisks indicate the p value (*p < 0.05; **p < 0.01; ***p < 0.001)

Next, we wondered if enhanced proliferation was the reason for the observed increase in Treg frequencies. Using Ki67^+^ as a marker for proliferating cells [28], we evaluated the proliferation rate of CD25^high^CD127^−^ Tregs over time (Fig. 3). No difference in Treg proliferation rates between healthy control patients and dialysis patients was detected. Furthermore, the proliferation rate during the treatment was not affected. A slight decrease has been observed two months after the treatment (at day 90) was compared to the baseline level for the dialysis patients. Taken together, the missing increase in Treg proliferation rate during the propionate phase in both study groups suggest that the Treg expansion is not due to an increased proliferation rate.

### Gut-homing regulatory T-cells are transiently decreased

Given the effect of sodium propionate on regulation and differentiation of T cell functions [29], we were wondering, if the propionate supplementation in some way changed the gut associated lymphoid tissue to expel the gut associated Tregs. This might explain the observed increase in Treg frequencies. Gut homing T-cells are characterized by the expression of the α4β7-integrine and CCR9 [30].

**Fig. 4:**
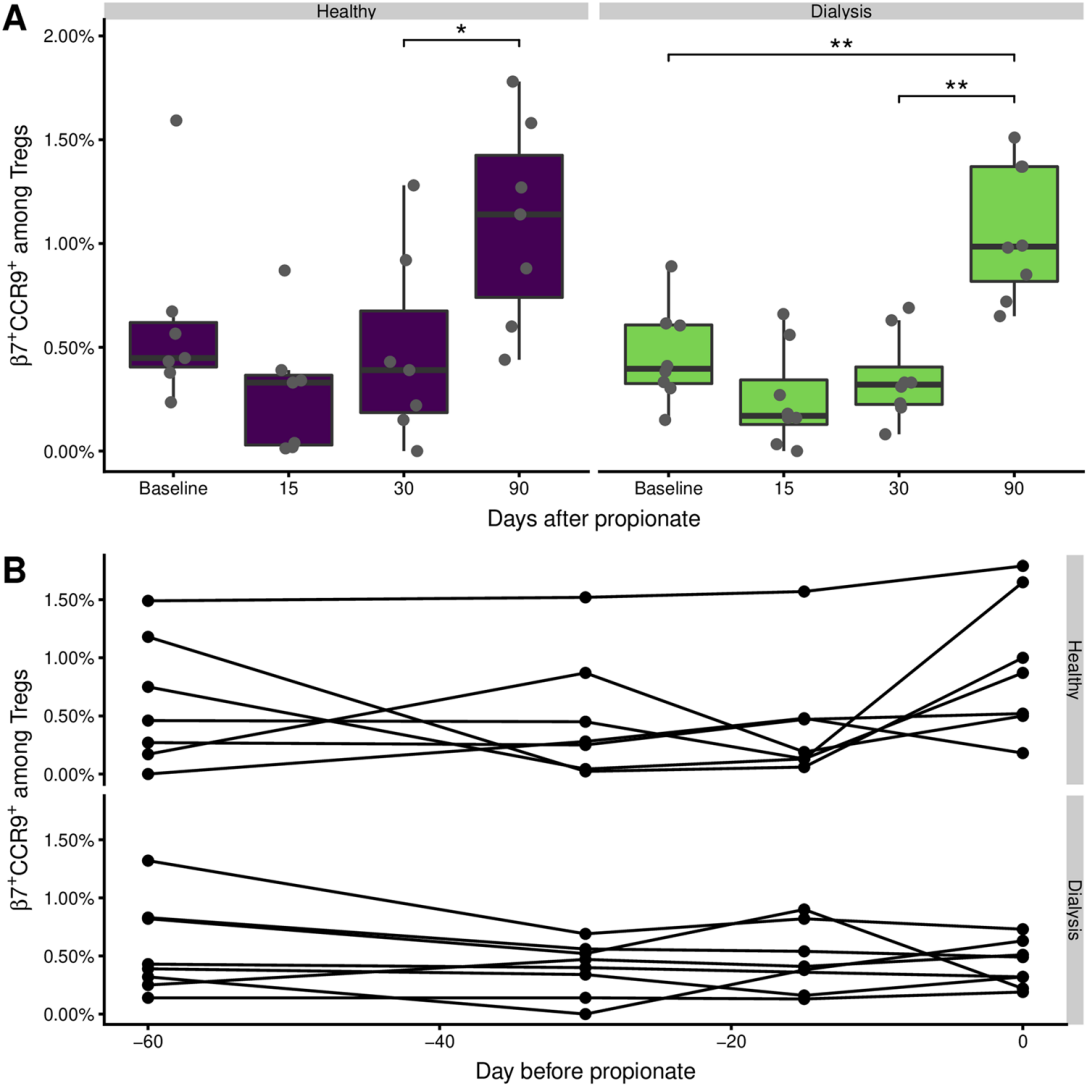
The frequency of gut homing Tregs is increased after propionate. Gut homing CD25^high^CD127^−^ Tregs were identified by the expression of α4β7^+^CCR9^+^ according to the gating strategy in Supplementary Fig. 2. The boxes represent the 25th, 50th, and 75th percentile and the whiskers represent the range of the observations excluding outliers. Each point signifies a single donor. Only significant differences are annotated. Asterisks indicate the p value (*p < 0.05; **p < 0.01; ***p < 0.001)

We found that the frequency of β7^+^ CCR9^+^ cells among Tregs decreased, albeit insignificantly, from the baseline level to day 15 of the propionate phase (Fig. 4a). This decrease was most prominent in the healthy control group, and was normalized at day 30. At the day 90 follow-up there was a significant increase compared to day 30, which was also different from baseline for the dialysis patients. This dynamic cannot be explained by baseline fluctuations (Fig. 4b), but rather by the migration of Treg into the intestine. Normalization in the level of gut-homing regulatory T-cells in peripheral blood might suggest their further expansion and saturation in gut tissue.

## Discussion

The low-grade chronic inflammation of ESRD patients is strongly associated with the increased risk of cardiovascular disease [1, 31, 32]. In fact, IL-6 levels are strong predictors of cardiovascular mortality in dialysis patients [33]. This makes the reduction of the inflammatory state an attractive aim [1]. Because of the accumulating evidence on the positive effect of SCFA, and particularly its major compound propionate, on inflammation and associated clinical symptoms, propionate is in this setting an appealing compound. In fact, Marzocco et al. demonstrated recently a significant decrease of inflammation markers, oxidative stress levels, and uremic toxins under propionate supplementation [6]. These changes were associated with an improvement in quality of life estimated by psycho-physical assessment.

While the works by Marzocco et al. analyzed variations in humoral immune factors, assessment of cellular immunity under propionate supplementation was previously performed by Haghikia et al. [17]. Using experimental autoimmune encephalomyelitis (EAE) as a model of T cell-mediated CNS autoimmunity the authors demonstrated that propionate supplementation led to expansion of Tregs and ameliorate the clinical course of disease [17]. In line with these data, we also detected increased frequency of peripheral Tregs during propionate supplementation in hemodialysis patients. 2 months after supplementation discontinuation, the frequencies of Tregs declined to the baseline level. A similar pattern of Treg kinetics was observed in healthy blood donors under propionate supplementation. Notably, we found no differences in the Treg frequencies between dialysis patients and healthy donors, neither in baseline nor after the propionate supplementation. These data are in accordance with other studies showing comparable Treg percentage proportions among CD4^+^ T-cells compared to healthy donors. The finding is, however, in contrast to other reports where an increased or decreased frequency of Tregs have been found in ESRD patients [34–36]. Likewise, conflicting observations regarding the functionality of Tregs in ESRD have been reported [37, 38]. In line with the kinetics of Tregs, we observed a significant decrease of CRP level during the propionate supplementation. CRP level increased to the level of baseline two months after therapy discontinuation.

The assessment of proliferative capacity and gut homing pattern of Tregs might enable conclusions on the mechanisms behind the observed Treg expansion under propionate supplementation. Of interest, we found no significant changes in the expression of Ki67 or β7/CCR9 on Tregs during the propionate phase. Therefore, our data suggest that neither the enhanced proliferation nor alterations in gut homing are responsible for the detected Treg expansion in peripheral blood. Furthermore, we did not observe any significant differences in the subset composition (naïve or memory Tregs) during the treatment, which might demonstrate influx of central memory Tregs from bone marrow niches or secondary lymphoid organs as a possible mechanism for T cell expansion [39].

The polarization of naïve T cells in peripheral blood toward certain T cell population represents an important mechanism in phenotypic T cell development [40]. In line with this, naïve T cell polarization toward Treg cells under propionate supplementation has been demonstrated in an EAE model [17]. The application of propionate to healthy donor naïve CD4^+^ T cells in vitro led to an increase in both the frequency of CD4^+^CD25^+^Foxp3^+^ cells and, to a lesser extent, the proliferation of differentiated Treg cells. Besides propionate, further SCFA such as butyrate and acetate have been reported to induce Treg expansion [29]. The increased extrathymic Treg differentiation has been previously demonstrated in a mouse model under SCFA supplementation [41]. De novo Treg generation in the periphery was related to SCFA of microbial origin (propionate or butyrate) and suggests that bacterial metabolites mediate communication between the commensal microbiota and the immunity [41]. The role of microbiota in the maintenance of the immune balance has been reported in numerous studies [42]. Alterations in gut microbiome are well described in patients with chronic kidney diseases [43]. This dysbiosis leads to insufficient generation of SCFA produced in in the distal small intestine and colon [6]. The lower level of SCFA is known to contribute to various autoimmune and systemic diseases such as inflammatory bowel diseases or multiple sclerosis [44]. By the supplementation of SCFA in ESRD patients, not only the level of SCFA can be restored, but the inflammation and dysbiosis can be alleviated and psycho-physical conditions can be improved as recently reported [6].

The positive anti-inflammatory effect provided by the SCFA supplementation, however, might counteract the composition and functionality of protective pathogen-reactive conventional T cells. As a model of functional activity of memory T cells, ex vivo recall antigen stimulation test can be performed [45]. Here, we analyzed the number and cytokine production of tetanus/diphtheria vaccine reactive T cells. Our data demonstrate no significant changes in the composition and functionality of these conventional T cells during or after propionate phase.

The gender distribution in ESRD group in the current study is heavily skewed towards males (80% males vs. 20% females). It has been shown, that SCFA levels are lower in men compared to women, but so is the general dietary fiber intake, indicating that diet plays an important role [46]. Similarly, neither gender nor age were shown to be confounding factors in comparison of SCFA levels in various gut related illness [47]. However, the distribution of gender within the present cohort could to some degree obscure the true effect of propionic acid.

Further limitations on the present study include the small patient number, a monocentric study design, and the lack of microbiome analysis. Nevertheless, the data presented here show that dietary supplements with SCFAs might have a beneficial effect on the elevated systemic inflammation of ESRD patients. The effect can be achieved through an expansion of circulating Tregs without affecting the antigen-specific memory response.

## Methods

### Study population and ethics statement

10 dialysis patients with ESRD of the Ruhr-University clinic Marien Hospital Herne and 7 healthy volunteers were enrolled in this self-control case series study (Table 2). The study protocol was approved by the ethical committee of the Ruhr-Universität Bochum (16-5840) and all participants gave written informed consent. Patients with an active or treated malignant disease in the last ten years, current immunosuppression, severe intestinal disease (e.g. inflammatory bowel disease), autoimmune diseases, and patients under antibiotics treatment during the last four weeks were excluded from the study.

**Table 2:** Cohort characteristics

Parameter	Healthy	ESRD patients
Number	7	10
Age (mean ± SD)	46.4 ± 17.1	73.8 ± 10.2
Age range	22 – 75	52 – 85
Gender female/male	4/3	2/8
Months of hemodialysis (mean ± SD)	n.a	66.7 ± 81.6

		n (%)
Causes of renal failure		
Diabetes mellitus	n.a	4 (40)
Arterial hypertension	n.a	1 (10)
Glomerulonephritis	n.a	2 (20)
Heart failure (cardiorenal syndrome)	n.a	1 (10)
Drug toxicity	n.a	1 (10)
Liver cirrhosis (hepatorenal syndrome)	n.a	1 (10)

		n (%)
Comorbidities		
Diabetes mellitus	0 (0)	9 (90)
Arterial hypertension	0 (0)	10 (100)
Obstructive sleep apnea syndrome	0 (0)	5 (50)
Chronic obstructive pulmonary disease	0 (0)	2 (20)
Peripheral artery disease	0 (0)	2 (10)
Coronary heart disease	0 (0)	4 (10)
Heart failure	0 (0)	3 (10)
Rheumatoid arthritis	0 (0)	1 (10)
Liver cirrhosis	0 (0)	1 (10)

*n.a.* not applicable

### Propionate dosage and sampling scheme

The study participants were followed for a total of 150 days and separated in the three phases (Supplementary Fig. 1). During the 30 days propionate phase, the study participants ingested 2 × 500 mg propionic acid per day. The last sample of the baseline phase (days − 60 to 0) was taken just prior to initiation of the propionate phase, and a measurement at day 90 constitute the follow-up phase.

### Preparation of PBMCs

Peripheral blood mononuclear cells (PBMCs) were prepared from whole blood by gradient centrifugation as previously described [48]. In brief, blood was collected in EDTA treated blood collection tubes (Sarstedt). Collected blood was pre-diluted in PBS/BSA (Gibco) at a 1:1 ratio and underlaid with 15 ml Ficoll-Paque Plus (GE Healthcare). Tubes were centrifuged at 800 g for 15 min at room temperature. Isolated PBMCs were washed twice with PBS/BSA.

### Stimulation with tetanus and diphtheria vaccine

1 ml of heparinized blood was incubated with 1 µl CD28 antibody (BD Biosciences, Clone CD28.2 RUO) and 50 µl tetanus/diphtheria vaccine (2 I.U. tetanus-toxoid and 0.2 I.U. diphtheria-toxoid; Sanofi Pasteur) 37 °C and 5% CO_2_. After 2 h of incubation Brefeldin-A (Sigma Aldrich) was added to a final concentration of 2 µg/ml. After a total of 6 h of incubation, the blood was lysed using RBC Lysis Buffer (BioLegend) for 10 min and pelleted at 280 g for 10 min at room temperature. The samples were washed twice with PBS/BSA and prepared for analysis by flow cytometry.

### Staining and flow cytometry

Antibody staining was performed at room temperature and in darkness as previously described [49]. Cells were acquired on a CytoFlex flow cytometer (Beckman Coulter). Quality control was performed daily using the recommended CytoFLEX Daily QC Fluorospheres (Beckman Coulter). No modification to the compensation matrices was required throughout the study.

For identification of Tregs, 30 million isolated PBMCs were centrifuged at 500*g* and incubated for 10 min in optimal dilutions of the antibodies in Table 3. Stained cells were subsequently prepared for intracellular staining using the Intracellular Fixation & Permeabilization Buffer Set (Thermo Fisher Scientific) as per manufacturer’s instructions. Fixed and permeable cells were stained for 30 min with optimal dilution of each antibody in Table 3. Stimulated cells subjected to the same protocol using optimal dilutions of the antibodies in Table 4.

**Table 3:** Antibodies used for Treg evaluation

Step	Antigen	Conjugate	Clone	Vendor
Surface	β7	PerCP/Cy5.5	FIB504	BioLegend
Surface	CCR7	Alexa Fluor 488	G043H7	BioLegend
Surface	CCR9	Brilliant Violet 421	L053E8	BioLegend
Surface	CD4	Alexa Fluor 700	OKT4	BioLegend
Surface	CD8	V500	RPA-T8	BD Biosciences
Surface	CD25	Pe-Cy7	2A3	BD Biosciences
Surface	CD45RA	Brilliant Violet 605	HI100	BioLegend
Surface	CD127	Brilliant Violet 650	A019D5	BioLegend
Surface	Live-Dead	eFL780		ThermoFisher Scientific
Intracellular	CD3	Brilliant Violet 785	OKT3	BioLegend
Intracellular	FoxP3	PE	PCH101	ThermoFisher Scientific
Intracellular	Helios	Alexa Fluor 647	22F6	BioLegend
Intracellular	Ki67	PE/Dazzle-594	Ki67	BioLegend

**Table 4:** Antibodies used for evaluation of T-cell activation

Step	Antigen	Conjugate	Clone	Vendor
Surface	CD4	Alexa-700	RPA-T4	eBioscience
Surface	CD8	V500	RPA-T8	BD Biosciences
Surface	Live-Dead	eFL780		ThermoFisher Scientific
Intracellular	IL-2	PE	MQ1-17H12	BioLegend
Intracellular	TNF-α	PerCPCy5.5	Mab11	BioLegend
Intracellular	CD154	Alexa-647	24–31	BioLegend
Intracellular	IL-17	Brilliant Violet 421	TC11-18H10.1	BioLegend
Intracellular	IFN-γ	Brilliant Violet 650	4S.B3	BioLegend
Intracellular	CD3	Brilliant Violet 785	OKT3	BioLegend

### Data analysis and statistics

The acquired flow cytometry data files were analyzed with the FlowJo (FlowJo LLC), version 10.5.3 and subsequently imported into R, version 3.3.5 for further analysis. Comparison of samples at different time points was performed using paired t test, while study groups were compared using an unpaired t test.

## Electronic supplementary material

Below is the link to the electronic supplementary material.

Supplementary file1 (DOCX 825 kb)
