# Supplementary material for: Propionate supplementation promotes the expansion of peripheral regulatory T-Cells in patients with end-stage renal disease
Source: J Nephrol. 2020 Mar 6;33(4):817–27. doi: 10.1007/s40620-019-00694-z (PMC7381474; doi:10.1007/s40620-019-00694-z)
Supplement: Supplementary file 1 — Supplementary file1 (DOCX 825 kb) [file 40620_2019_694_MOESM1_ESM.docx]

Propionate supplementation promotes the expansion of peripheral regulatory T-Cells in patients with end-stage renal disease – Supplementary information

Fabian Meyer^1^, Felix Seibert MD^1^, Mikalai Nienen PhD^1^, Marius Welzel M.Sc.^2^, Daniela Beisser PhD^2^, Frederic Bauer MD^1^, Benjamin Rohn MD^1^, Timm H Westhoff MD^1^, Ulrik Stervbo PhD^1*^, Nina Babel MD^1,3*^

^1^Marienhospital Herne – Universitätsklinikum der Ruhr-Universität Bochum, Medical Department I, Herne, Germany

^2^University of Duisburg-Essen; Biodiversity, Essen, Germany

^3^Charité – Universitätsmedizin Berlin, Corporate Member of Freie Universität Berlin, Humboldt-Universität zu Berlin, and Berlin Institute of Health, Berlin-Brandenburg Center for Regenerative Therapies, Berlin, Germany

^*^Equally contributing

## Correspondence

Ulrik Stervbo

Centre for Translational Medicine – Medical Clinic I

Universitätsklinikum der Ruhr-Universität Bochum, Ruhr-Universität Bochum

Hölkeskampring 40

44623 Herne

Germany

E-mail: Ulrik.stervbo@elisabethgruppe.de

Phone: +49 2323 499 1028

Nina Babel

Centre for Translational Medicine – Medical Clinic I

Universitätsklinikum der Ruhr-Universität Bochum, Ruhr-Universität Bochum

Hölkeskampring 40

44623 Herne

Germany

E-mail: Nina.babel@elisabethgruppe.de

Phone: +49 2323 499 1028


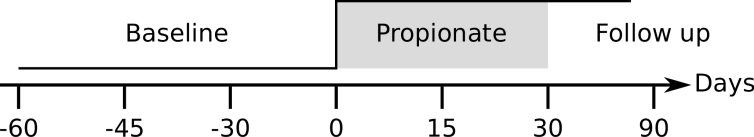


## Supplementary Figure 1. Study outline

The study participants were followed for a total of 150 days. The sampling frequency, marked by ticks, was 15 to 30 days. The baseline of cellular frequencies was established by following the participants for 60 days before initiation of a dietary supplement with propionate (baseline phase; days –60 to 0). The phase of supplementation lasted 30 days (propionate phase; gray box) with a single post-propionate follow up point at day 90 (follow up phase). Each tick represents a sampling event.


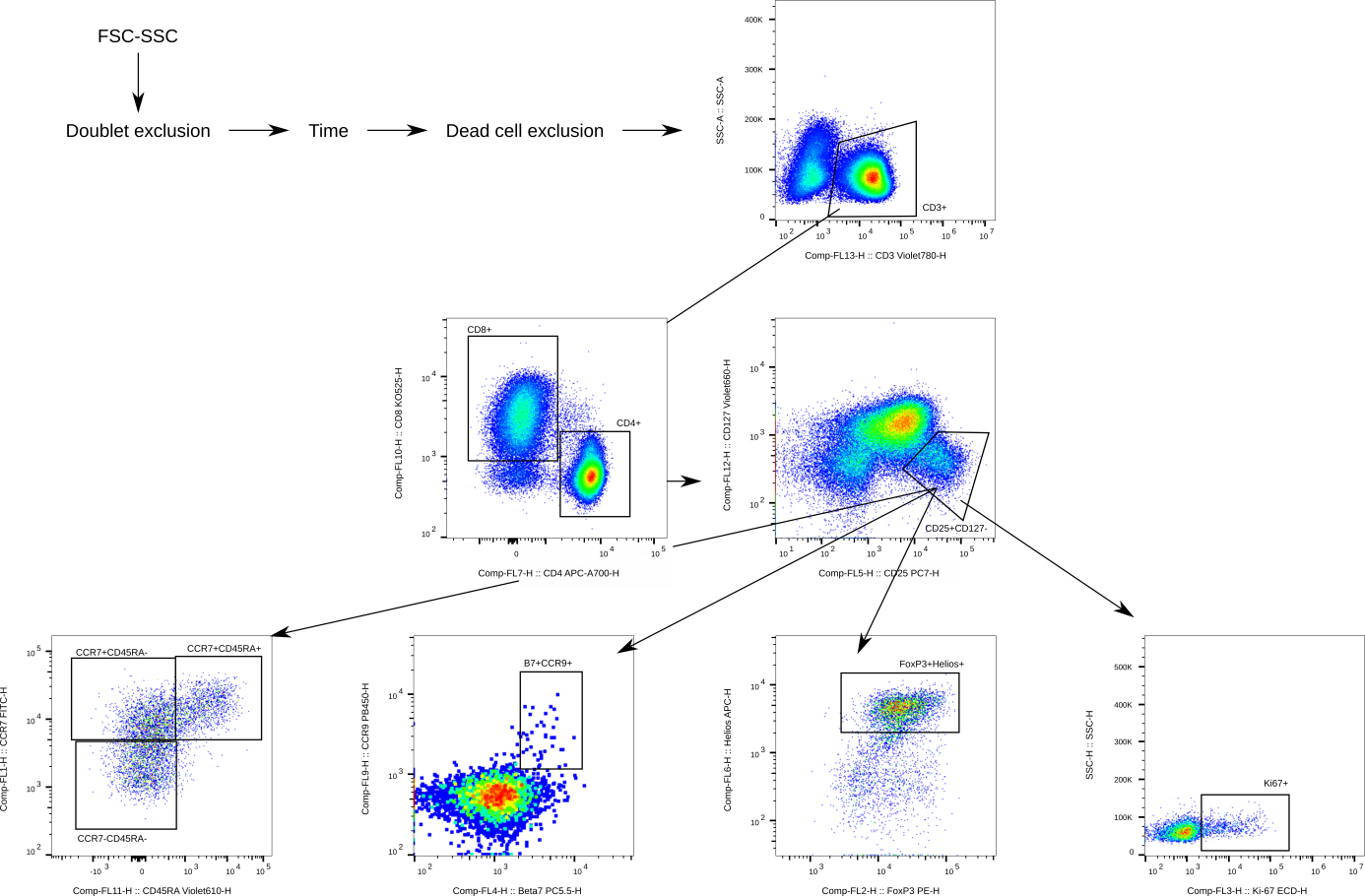


## Supplementary Figure 2. Gating strategy – CD25^high^CD127^–^ Tregs

Tregs were identified as CD25^high^CD127^–^ by *ex vivo* flow cytometry. The presented strategy excludes doublet exclusion, dead cells, and a time gate.


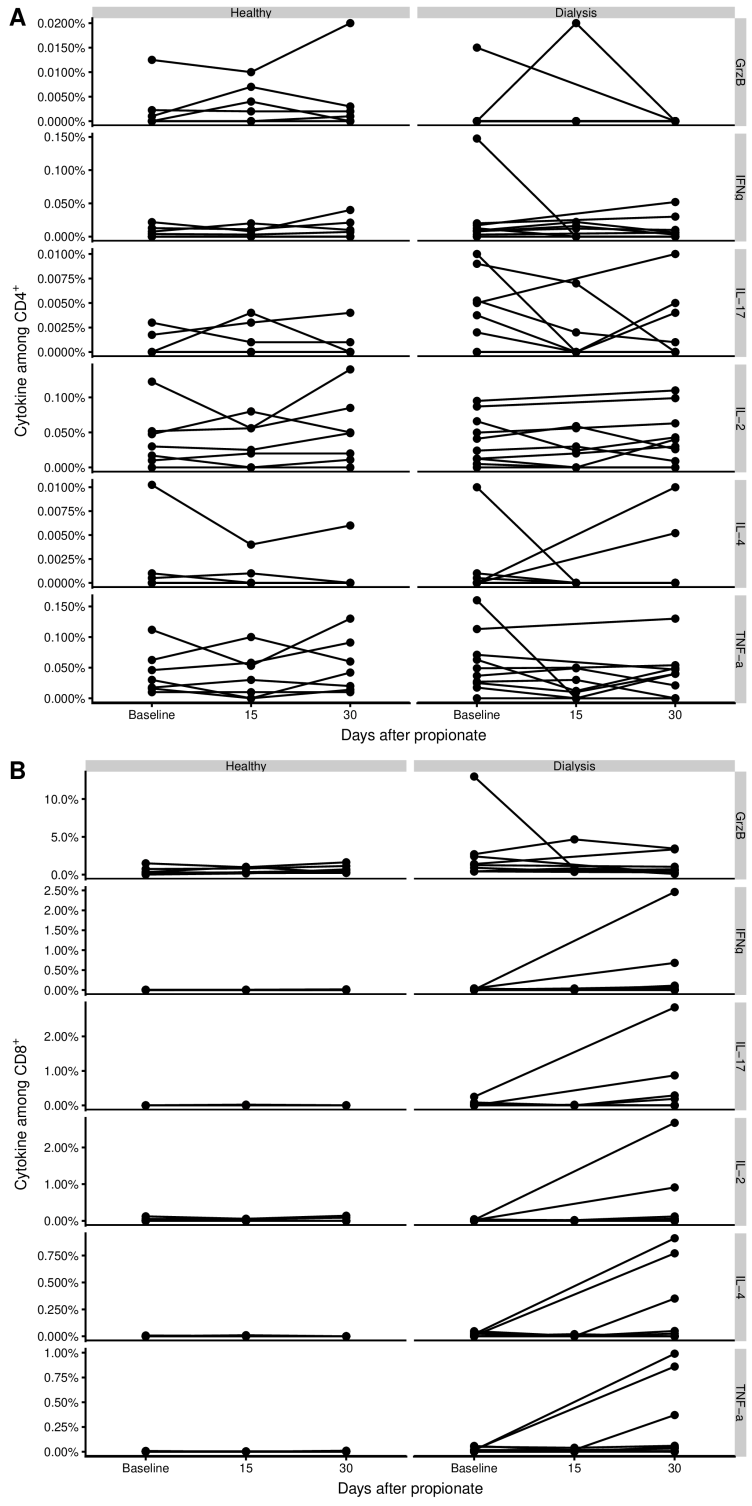


## Supplementary Figure 3. T-cell functionality does not change with propionate

T-cells activated by the recall antigen of the Tetanus-Diphtheria vaccine were analyzed by flow cytometry during the propionate phase according to the gating strategy in Supplementary Figure 4. A) The frequency of granzyme-B (GrzB), IFN-γ, IL-17, IL-2, IL-4, and TNF-α among CD4^+^ T-cells. B) The frequency of the cytokines granzyme-B (GrzB), IFN-γ, IL-17, IL-2, IL-4, and TNF-α among CD8^+^ T-cells. Each line indicates a study participant. No significant differences were observed.


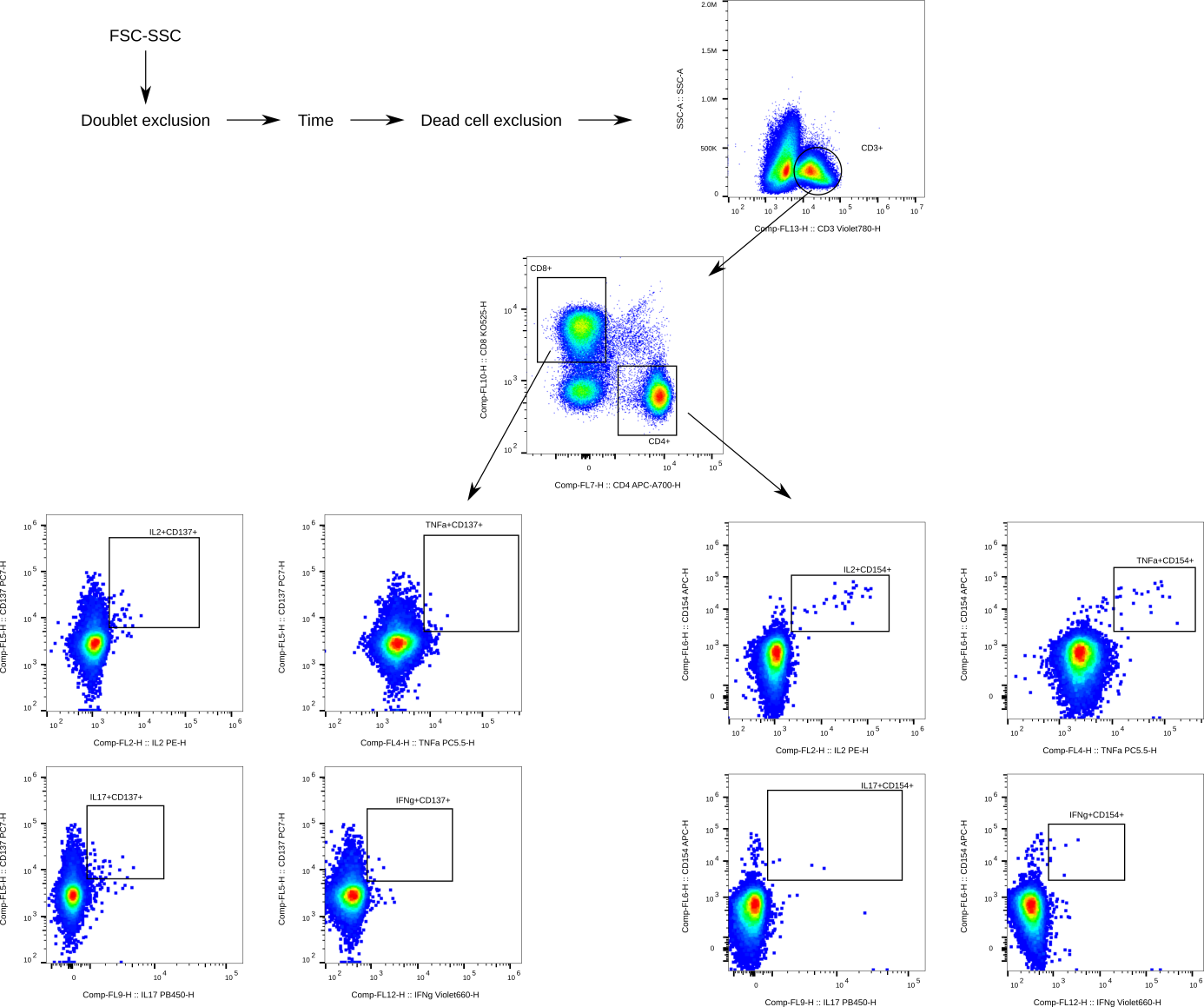


## Supplementary Figure 4. Gating strategy – Tetanus-Diphtheria specific T-cells

T-cells activated by the recall antigen of the Tetanus-Diphtheria vaccine were analyzed by flow cytometry. The presented strategy excludes doublet exclusion, dead cells, and a time gate.
